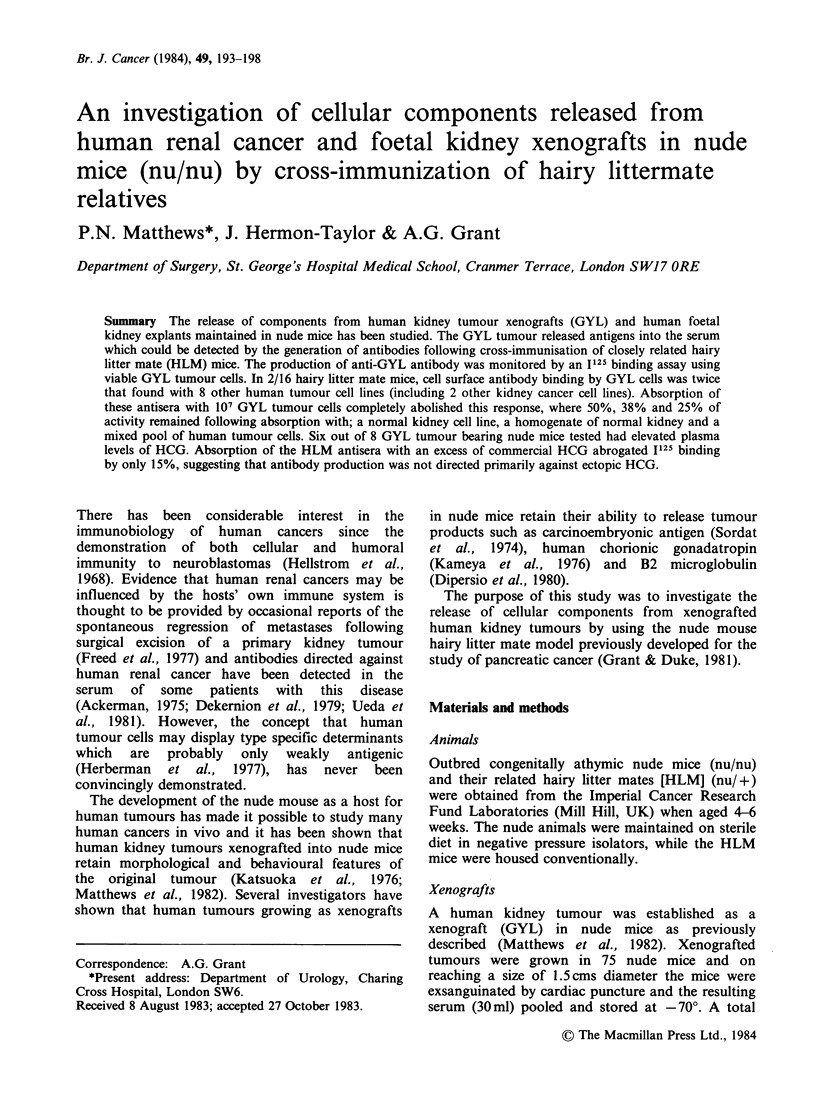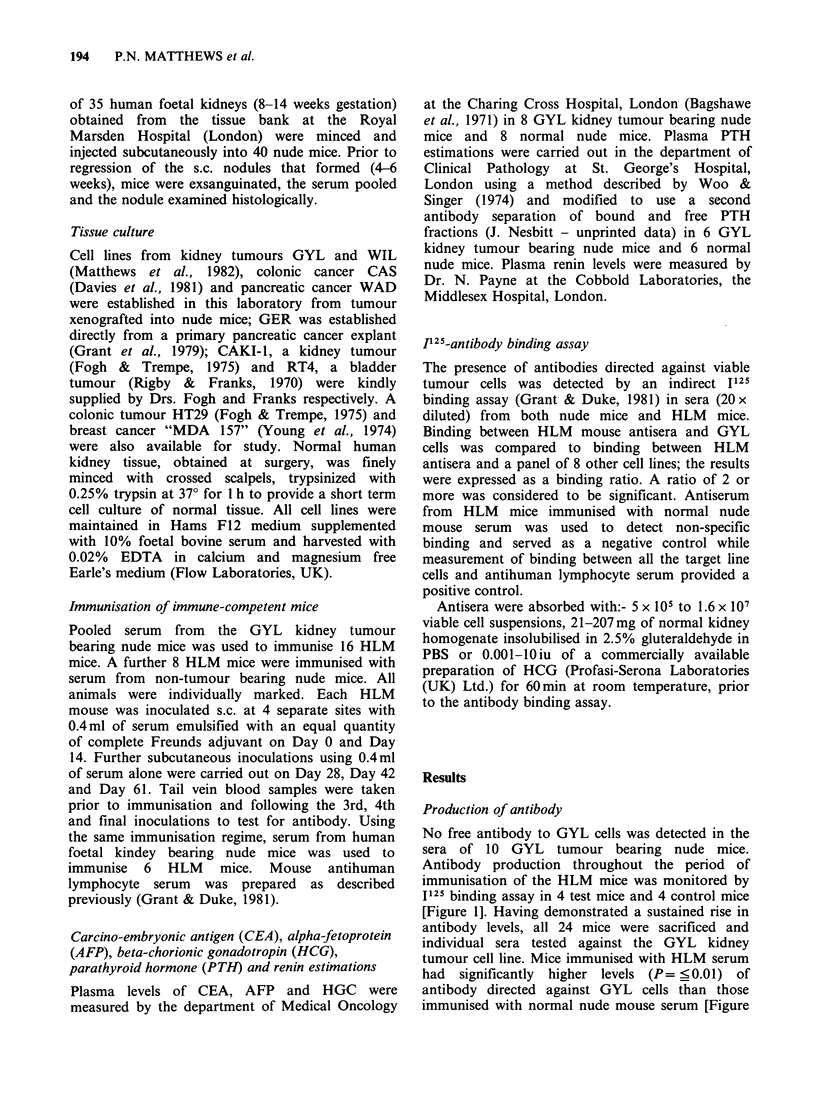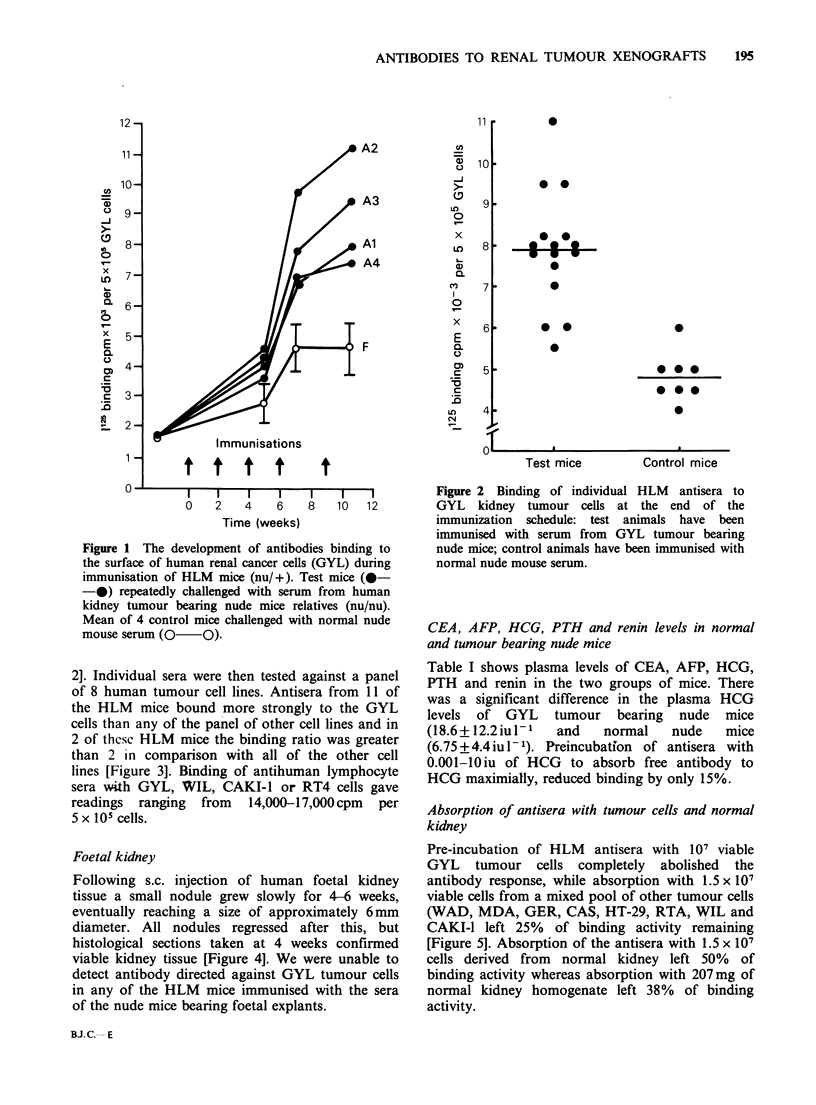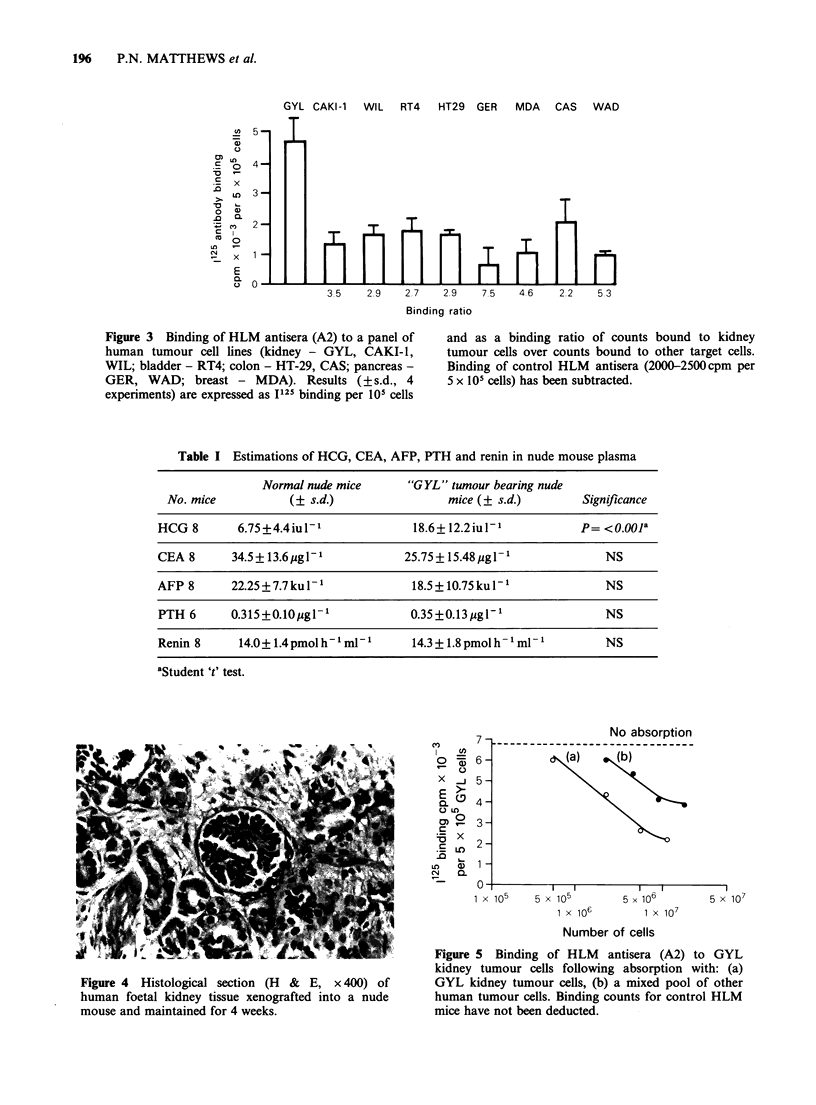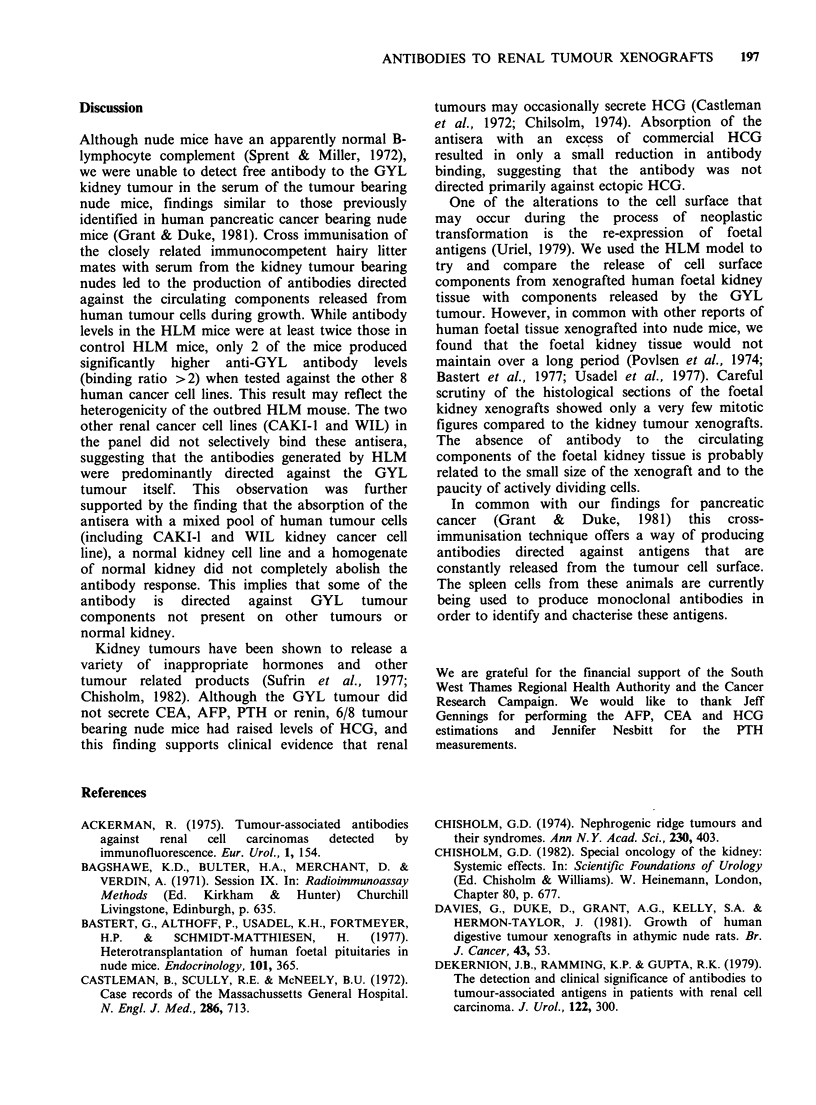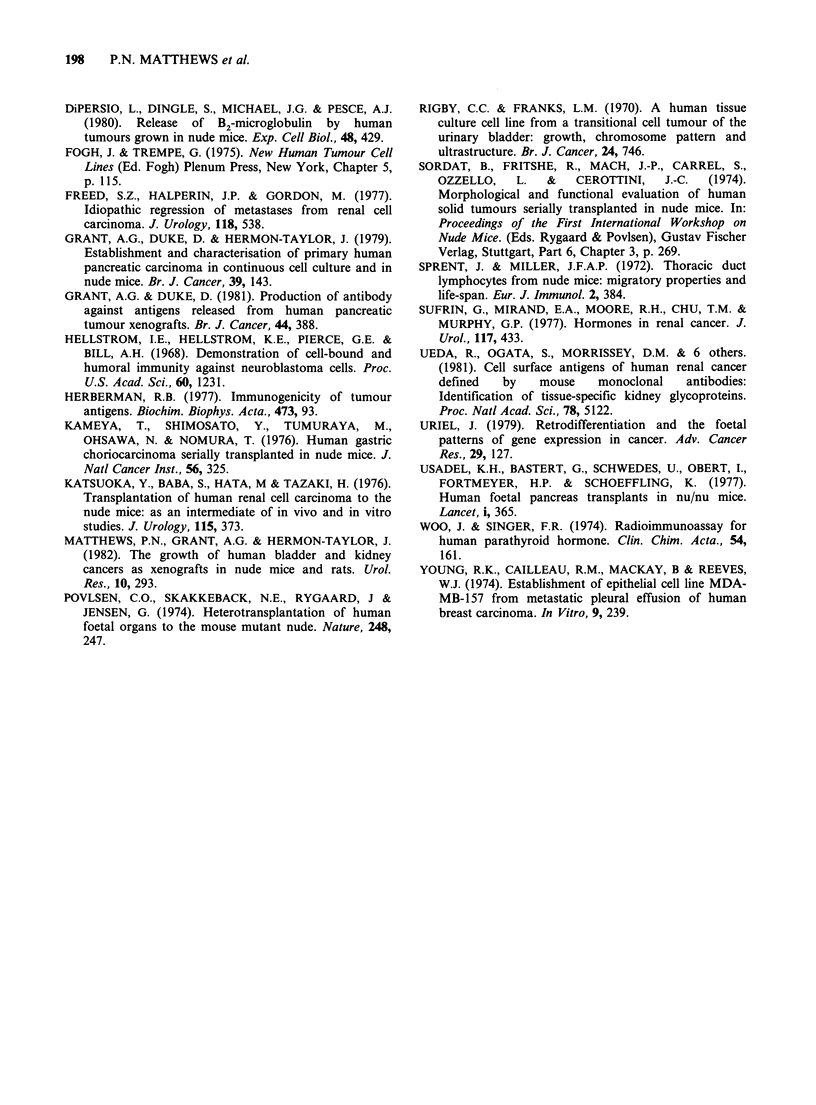# An investigation of cellular components released from human renal cancer and foetal kidney xenografts in nude mice (nu/nu) by cross-immunization of hairy littermate relatives.

**DOI:** 10.1038/bjc.1984.31

**Published:** 1984-02

**Authors:** P. N. Matthews, J. Hermon-Taylor, A. G. Grant

## Abstract

**Images:**


Br. J. Cancer (1984), 49, 193–198

### An investigation of cellular components released from human renal cancer and foetal kidney xenografts in nude mice (nu/nu) by cross-immunization of hairy littermate relatives

P.N. Matthews*, J. Hermon-Taylor & A.G. Grant

*Department of Surgery, St. George's Hospital Medical School, Cranmer Terrace, London SW17 0RE*

**Summary** The release of components from human kidney tumour xenografts (GYL) and human foetal kidney explants maintained in nude mice has been studied. The GYL tumour released antigens into the serum which could be detected by the generation of antibodies following cross-immunisation of closely related hairy litter mate (HLM) mice. The production of anti-GYL antibody was monitored by an $I^{125}$ binding assay using viable GYL tumour cells. In 2/16 hairy litter mate mice, cell surface antibody binding by GYL cells was twice that found with 8 other human tumour cell lines (including 2 other kidney cancer cell lines). Absorption of these antisera with $10^7$ GYL tumour cells completely abolished this response, where 50%, 38% and 25% of activity remained following absorption with; a normal kidney cell line, a homogenate of normal kidney and a mixed pool of human tumour cells. Six out of 8 GYL tumour bearing nude mice tested had elevated plasma levels of HCG. Absorption of the HLM antisera with an excess of commercial HCG abrogated $I^{125}$ binding by only 15%, suggesting that antibody production was not directed primarily against ectopic HCG.

There has been considerable interest in the immunobiology of human cancers since the demonstration of both cellular and humoral immunity to neuroblastomas (Hellstrom *et al.*, 1968). Evidence that human renal cancers may be influenced by the hosts' own immune system is thought to be provided by occasional reports of the spontaneous regression of metastases following surgical excision of a primary kidney tumour (Freed *et al.*, 1977) and antibodies directed against human renal cancer have been detected in the serum of some patients with this disease (Ackerman, 1975; Dekernion *et al.*, 1979; Ueda *et al.*, 1981). However, the concept that human tumour cells may display type specific determinants which are probably only weakly antigenic (Herberman *et al.*, 1977), has never been convincingly demonstrated.

The development of the nude mouse as a host for human tumours has made it possible to study many human cancers in vivo and it has been shown that human kidney tumours xenografted into nude mice retain morphological and behavioural features of the original tumour (Katsuoka *et al.*, 1976; Matthews *et al.*, 1982). Several investigators have shown that human tumours growing as xenografts in nude mice retain their ability to release tumour products such as carcinoembryonic antigen (Sordat *et al.*, 1974), human chorionic gonadatropin (Kameya *et al.*, 1976) and B2 microglobulin (Dipersio *et al.*, 1980).

The purpose of this study was to investigate the release of cellular components from xenografted human kidney tumours by using the nude mouse hairy litter mate model previously developed for the study of pancreatic cancer (Grant & Duke, 1981).

Correspondence: A.G. Grant
*Present address: Department of Urology, Charing Cross Hospital, London SW6.
Received 8 August 1983; accepted 27 October 1983.

### Materials and methods

### *Animals*

Outbred congenitally athymic nude mice (nu/nu) and their related hairy litter mates [HLM] (nu/+) were obtained from the Imperial Cancer Research Fund Laboratories (Mill Hill, UK) when aged 4–6 weeks. The nude animals were maintained on sterile diet in negative pressure isolators, while the HLM mice were housed conventionally.

### *Xenografts*

A human kidney tumour was established as a xenograft (GYL) in nude mice as previously described (Matthews *et al.*, 1982). Xenografted tumours were grown in 75 nude mice and on reaching a size of 1.5 cms diameter the mice were exsanguinated by cardiac puncture and the resulting serum (30 ml) pooled and stored at −70°. A total

© The Macmillan Press Ltd., 1984

of 35 human foetal kidneys (8–14 weeks gestation) obtained from the tissue bank at the Royal Marsden Hospital (London) were minced and injected subcutaneously into 40 nude mice. Prior to regression of the s.c. nodules that formed (4–6 weeks), mice were exsanguinated, the serum pooled and the nodule examined histologically.

*Tissue culture*

Cell lines from kidney tumours GYL and WIL (Matthews *et al.*, 1982), colonic cancer CAS (Davies *et al.*, 1981) and pancreatic cancer WAD were established in this laboratory from tumour xenografted into nude mice; GER was established directly from a primary pancreatic cancer explant (Grant *et al.*, 1979); CAKI-1, a kidney tumour (Fogh & Trempe, 1975) and RT4, a bladder tumour (Rigby & Franks, 1970) were kindly supplied by Drs. Fogh and Franks respectively. A colonic tumour HT29 (Fogh & Trempe, 1975) and breast cancer "MDA 157" (Young *et al.*, 1974) were also available for study. Normal human kidney tissue, obtained at surgery, was finely minced with crossed scalpels, trypsinized with 0.25% trypsin at 37° for 1 h to provide a short term cell culture of normal tissue. All cell lines were maintained in Hams F12 medium supplemented with 10% foetal bovine serum and harvested with 0.02% EDTA in calcium and magnesium free Earle's medium (Flow Laboratories, UK).

*Immunisation of immune-competent mice*

Pooled serum from the GYL kidney tumour bearing nude mice was used to immunise 16 HLM mice. A further 8 HLM mice were immunised with serum from non-tumour bearing nude mice. All animals were individually marked. Each HLM mouse was inoculated s.c. at 4 separate sites with 0.4 ml of serum emulsified with an equal quantity of complete Freunds adjuvant on Day 0 and Day 14. Further subcutaneous inoculations using 0.4 ml of serum alone were carried out on Day 28, Day 42 and Day 61. Tail vein blood samples were taken prior to immunisation and following the 3rd, 4th and final inoculations to test for antibody. Using the same immunisation regime, serum from human foetal kindey bearing nude mice was used to immunise 6 HLM mice. Mouse antihuman lymphocyte serum was prepared as described previously (Grant & Duke, 1981).

*Carcino-embryonic antigen (CEA), alpha-fetoprotein (AFP), beta-chorionic gonadotropin (HCG), parathyroid hormone (PTH) and renin estimations*

Plasma levels of CEA, AFP and HGC were measured by the department of Medical Oncology at the Charing Cross Hospital, London (Bagshawe *et al.*, 1971) in 8 GYL kidney tumour bearing nude mice and 8 normal nude mice. Plasma PTH estimations were carried out in the department of Clinical Pathology at St. George's Hospital, London using a method described by Woo & Singer (1974) and modified to use a second antibody separation of bound and free PTH fractions (J. Nesbitt – unprinted data) in 6 GYL kidney tumour bearing nude mice and 6 normal nude mice. Plasma renin levels were measured by Dr. N. Payne at the Cobbold Laboratories, the Middlesex Hospital, London.

*$I^{125}$-antibody binding assay*

The presence of antibodies directed against viable tumour cells was detected by an indirect $I^{125}$ binding assay (Grant & Duke, 1981) in sera (20 × diluted) from both nude mice and HLM mice. Binding between HLM mouse antisera and GYL cells was compared to binding between HLM antisera and a panel of 8 other cell lines; the results were expressed as a binding ratio. A ratio of 2 or more was considered to be significant. Antiserum from HLM mice immunised with normal nude mouse serum was used to detect non-specific binding and served as a negative control while measurement of binding between all the target line cells and antihuman lymphocyte serum provided a positive control.

Antisera were absorbed with:- $5 \times 10^5$ to $1.6 \times 10^7$ viable cell suspensions, 21–207 mg of normal kidney homogenate insolubilised in 2.5% gluteraldehyde in PBS or 0.001–10 iu of a commercially available preparation of HCG (Profasi-Serona Laboratories (UK) Ltd.) for 60 min at room temperature, prior to the antibody binding assay.

### Results

*Production of antibody*

No free antibody to GYL cells was detected in the sera of 10 GYL tumour bearing nude mice. Antibody production throughout the period of immunisation of the HLM mice was monitored by $I^{125}$ binding assay in 4 test mice and 4 control mice [Figure 1]. Having demonstrated a sustained rise in antibody levels, all 24 mice were sacrificed and individual sera tested against the GYL kidney tumour cell line. Mice immunised with HLM serum had significantly higher levels ($P = \leq 0.01$) of antibody directed against GYL cells than those immunised with normal nude mouse serum [Figure

**Figure 1** The development of antibodies binding to the surface of human renal cancer cells (GYL) during immunisation of HLM mice (nu/+). Test mice (●——●) repeatedly challenged with serum from human kidney tumour bearing nude mice relatives (nu/nu). Mean of 4 control mice challenged with normal nude mouse serum (○——○).

**Figure 2** Binding of individual HLM antisera to GYL kidney tumour cells at the end of the immunization schedule: test animals have been immunised with serum from GYL tumour bearing nude mice; control animals have been immunised with normal nude mouse serum.

2]. Individual sera were then tested against a panel of 8 human tumour cell lines. Antisera from 11 of the HLM mice bound more strongly to the GYL cells than any of the panel of other cell lines and in 2 of these HLM mice the binding ratio was greater than 2 in comparison with all of the other cell lines [Figure 3]. Binding of antihuman lymphocyte sera with GYL, WIL, CAKI-1 or RT4 cells gave readings ranging from 14,000–17,000 cpm per $5\times10^5$ cells.

*Foetal kidney*

Following s.c. injection of human foetal kidney tissue a small nodule grew slowly for 4–6 weeks, eventually reaching a size of approximately 6 mm diameter. All nodules regressed after this, but histological sections taken at 4 weeks confirmed viable kidney tissue [Figure 4]. We were unable to detect antibody directed against GYL tumour cells in any of the HLM mice immunised with the sera of the nude mice bearing foetal explants.

*CEA, AFP, HCG, PTH and renin levels in normal and tumour bearing nude mice*

Table I shows plasma levels of CEA, AFP, HCG, PTH and renin in the two groups of mice. There was a significant difference in the plasma HCG levels of GYL tumour bearing nude mice ($18.6\pm12.2$ iu $l^{-1}$ and normal nude mice ($6.75\pm4.4$ iu $l^{-1}$). Preincubation of antisera with 0.001–10 iu of HCG to absorb free antibody to HCG maximially, reduced binding by only 15%.

*Absorption of antisera with tumour cells and normal kidney*

Pre-incubation of HLM antisera with $10^7$ viable GYL tumour cells completely abolished the antibody response, while absorption with $1.5\times10^7$ viable cells from a mixed pool of other tumour cells (WAD, MDA, GER, CAS, HT-29, RTA, WIL and CAKI-1 left 25% of binding activity remaining [Figure 5]. Absorption of the antisera with $1.5\times10^7$ cells derived from normal kidney left 50% of binding activity whereas absorption with 207 mg of normal kidney homogenate left 38% of binding activity.

**Figure 3** Binding of HLM antisera (A2) to a panel of human tumour cell lines (kidney – GYL, CAKI-1, WIL; bladder – RT4; colon – HT-29, CAS; pancreas – GER, WAD; breast – MDA). Results (±s.d., 4 experiments) are expressed as $I^{125}$ binding per $10^5$ cells and as a binding ratio of counts bound to kidney tumour cells over counts bound to other target cells. Binding of control HLM antisera (2000–2500 cpm per $5 \times 10^5$ cells) has been subtracted.

**Table I** Estimations of HCG, CEA, AFP, PTH and renin in nude mouse plasma

| *No. mice* | *Normal nude mice* (± *s.d.*) | *"GYL" tumour bearing nude mice* (± *s.d.*) | *Significance* |
|---|---|---|---|
| HCG 8 | $6.75 \pm 4.4\,iu\,l^{-1}$ | $18.6 \pm 12.2\,iu\,l^{-1}$ | *P= <0.001*[a] |
| CEA 8 | $34.5 \pm 13.6\,\mu g\,l^{-1}$ | $25.75 \pm 15.48\,\mu g\,l^{-1}$ | NS |
| AFP 8 | $22.25 \pm 7.7\,ku\,l^{-1}$ | $18.5 \pm 10.75\,ku\,l^{-1}$ | NS |
| PTH 6 | $0.315 \pm 0.10\,\mu g\,l^{-1}$ | $0.35 \pm 0.13\,\mu g\,l^{-1}$ | NS |
| Renin 8 | $14.0 \pm 1.4\,pmol\,h^{-1}\,ml^{-1}$ | $14.3 \pm 1.8\,pmol\,h^{-1}\,ml^{-1}$ | NS |

[a]Student '*t*' test.

**Figure 4** Histological section (H & E, ×400) of human foetal kidney tissue xenografted into a nude mouse and maintained for 4 weeks.

**Figure 5** Binding of HLM antisera (A2) to GYL kidney tumour cells following absorption with: (a) GYL kidney tumour cells, (b) a mixed pool of other human tumour cells. Binding counts for control HLM mice have not been deducted.

### Discussion

Although nude mice have an apparently normal B-lymphocyte complement (Sprent & Miller, 1972), we were unable to detect free antibody to the GYL kidney tumour in the serum of the tumour bearing nude mice, findings similar to those previously identified in human pancreatic cancer bearing nude mice (Grant & Duke, 1981). Cross immunisation of the closely related immunocompetent hairy litter mates with serum from the kidney tumour bearing nudes led to the production of antibodies directed against the circulating components released from human tumour cells during growth. While antibody levels in the HLM mice were at least twice those in control HLM mice, only 2 of the mice produced significantly higher anti-GYL antibody levels (binding ratio >2) when tested against the other 8 human cancer cell lines. This result may reflect the heterogenicity of the outbred HLM mouse. The two other renal cancer cell lines (CAKI-1 and WIL) in the panel did not selectively bind these antisera, suggesting that the antibodies generated by HLM were predominantly directed against the GYL tumour itself. This observation was further supported by the finding that the absorption of the antisera with a mixed pool of human tumour cells (including CAKI-1 and WIL kidney cancer cell line), a normal kidney cell line and a homogenate of normal kidney did not completely abolish the antibody response. This implies that some of the antibody is directed against GYL tumour components not present on other tumours or normal kidney.

Kidney tumours have been shown to release a variety of inappropriate hormones and other tumour related products (Sufrin *et al.*, 1977; Chisholm, 1982). Although the GYL tumour did not secrete CEA, AFP, PTH or renin, 6/8 tumour bearing nude mice had raised levels of HCG, and this finding supports clinical evidence that renal tumours may occasionally secrete HCG (Castleman *et al.*, 1972; Chilsolm, 1974). Absorption of the antisera with an excess of commercial HCG resulted in only a small reduction in antibody binding, suggesting that the antibody was not directed primarily against ectopic HCG.

One of the alterations to the cell surface that may occur during the process of neoplastic transformation is the re-expression of foetal antigens (Uriel, 1979). We used the HLM model to try and compare the release of cell surface components from xenografted human foetal kidney tissue with components released by the GYL tumour. However, in common with other reports of human foetal tissue xenografted into nude mice, we found that the foetal kidney tissue would not maintain over a long period (Povlsen *et al.*, 1974; Bastert *et al.*, 1977; Usadel *et al.*, 1977). Careful scrutiny of the histological sections of the foetal kidney xenografts showed only a very few mitotic figures compared to the kidney tumour xenografts. The absence of antibody to the circulating components of the foetal kidney tissue is probably related to the small size of the xenograft and to the paucity of actively dividing cells.

In common with our findings for pancreatic cancer (Grant & Duke, 1981) this cross-immunisation technique offers a way of producing antibodies directed against antigens that are constantly released from the tumour cell surface. The spleen cells from these animals are currently being used to produce monoclonal antibodies in order to identify and chacterise these antigens.

We are grateful for the financial support of the South West Thames Regional Health Authority and the Cancer Research Campaign. We would like to thank Jeff Gennings for performing the AFP, CEA and HCG estimations and Jennifer Nesbitt for the PTH measurements.

### References

ACKERMAN, R. (1975). Tumour-associated antibodies against renal cell carcinomas detected by immunofluorescence. *Eur. Urol.*, **1**, 154.

BAGSHAWE, K.D., BULTER, H.A., MERCHANT, D. & VERDIN, A. (1971). Session IX. In: *Radioimmunoassay Methods* (Ed. Kirkham & Hunter) Churchill Livingstone, Edinburgh, p. 635.

BASTERT, G., ALTHOFF, P., USADEL, K.H., FORTMEYER, H.P. & SCHMIDT-MATTHIESEN, H. (1977). Heterotransplantation of human foetal pituitaries in nude mice. *Endocrinology*, **101**, 365.

CASTLEMAN, B., SCULLY, R.E. & McNEELY, B.U. (1972). Case records of the Massachussetts General Hospital. *N. Engl. J. Med.*, **286**, 713.

CHISHOLM, G.D. (1974). Nephrogenic ridge tumours and their syndromes. *Ann N.Y. Acad. Sci.*, **230**, 403.

CHISHOLM, G.D. (1982). Special oncology of the kidney: Systemic effects. In: *Scientific Foundations of Urology* (Ed. Chisholm & Williams). W. Heinemann, London, Chapter 80, p. 677.

DAVIES, G., DUKE, D., GRANT, A.G., KELLY, S.A. & HERMON-TAYLOR, J. (1981). Growth of human digestive tumour xenografts in athymic nude rats. *Br. J. Cancer*, **43**, 53.

DEKERNION, J.B., RAMMING, K.P. & GUPTA, R.K. (1979). The detection and clinical significance of antibodies to tumour-associated antigens in patients with renal cell carcinoma. *J. Urol.*, **122**, 300.

DiPERSIO, L., DINGLE, S., MICHAEL, J.G. & PESCE, A.J. (1980). Release of $B_2$-microglobulin by human tumours grown in nude mice. *Exp. Cell Biol.*, **48**, 429.

FOGH, J. & TREMPE, G. (1975). *New Human Tumour Cell Lines* (Ed. Fogh) Plenum Press, New York, Chapter 5, p. 115.

FREED, S.Z., HALPERIN, J.P. & GORDON, M. (1977). Idiopathic regression of metastases from renal cell carcinoma. *J. Urology*, **118**, 538.

GRANT, A.G., DUKE, D. & HERMON-TAYLOR, J. (1979). Establishment and characterisation of primary human pancreatic carcinoma in continuous cell culture and in nude mice. *Br. J. Cancer*, **39**, 143.

GRANT, A.G. & DUKE, D. (1981). Production of antibody against antigens released from human pancreatic tumour xenografts. *Br. J. Cancer*, **44**, 388.

HELLSTROM, I.E., HELLSTROM, K.E., PIERCE, G.E. & BILL, A.H. (1968). Demonstration of cell-bound and humoral immunity against neuroblastoma cells. *Proc. U.S. Acad. Sci.*, **60**, 1231.

HERBERMAN, R.B. (1977). Immunogenicity of tumour antigens. *Biochim. Biophys. Acta.*, **473**, 93.

KAMEYA, T., SHIMOSATO, Y., TUMURAYA, M., OHSAWA, N. & NOMURA, T. (1976). Human gastric choriocarcinoma serially transplanted in nude mice. *J. Natl Cancer Inst.*, **56**, 325.

KATSUOKA, Y., BABA, S., HATA, M & TAZAKI, H. (1976). Transplantation of human renal cell carcinoma to the nude mice: as an intermediate of in vivo and in vitro studies. *J. Urology*, **115**, 373.

MATTHEWS, P.N., GRANT, A.G. & HERMON-TAYLOR, J. (1982). The growth of human bladder and kidney cancers as xenografts in nude mice and rats. *Urol. Res.*, **10**, 293.

POVLSEN, C.O., SKAKKEBACK, N.E., RYGAARD, J & JENSEN, G. (1974). Heterotransplantation of human foetal organs to the mouse mutant nude. *Nature*, **248**, 247.

RIGBY, C.C. & FRANKS, L.M. (1970). A human tissue culture cell line from a transitional cell tumour of the urinary bladder: growth, chromosome pattern and ultrastructure. *Br. J. Cancer*, **24**, 746.

SORDAT, B., FRITSHE, R., MACH, J.-P., CARREL, S., OZZELLO, L. & CEROTTINI, J.-C. (1974). Morphological and functional evaluation of human solid tumours serially transplanted in nude mice. In: *Proceedings of the First International Workshop on Nude Mice.* (Eds. Rygaard & Povlsen), Gustav Fischer Verlag, Stuttgart, Part 6, Chapter 3, p. 269.

SPRENT, J. & MILLER, J.F.A.P. (1972). Thoracic duct lymphocytes from nude mice: migratory properties and life-span. *Eur. J. Immunol.* **2**, 384.

SUFRIN, G., MIRAND, E.A., MOORE, R.H., CHU, T.M. & MURPHY, G.P. (1977). Hormones in renal cancer. *J. Urol.*, **117**, 433.

UEDA, R., OGATA, S., MORRISSEY, D.M. & 6 others. (1981). Cell surface antigens of human renal cancer defined by mouse monoclonal antibodies: Identification of tissue-specific kidney glycoproteins. *Proc. Natl Acad. Sci.*, **78**, 5122.

URIEL, J. (1979). Retrodifferentiation and the foetal patterns of gene expression in cancer. *Adv. Cancer Res.*, **29**, 127.

USADEL, K.H., BASTERT, G., SCHWEDES, U., OBERT, I., FORTMEYER, H.P. & SCHOEFFLING, K. (1977). Human foetal pancreas transplants in nu/nu mice. *Lancet*, **i**, 365.

WOO, J. & SINGER, F.R. (1974). Radioimmunoassay for human parathyroid hormone. *Clin. Chim. Acta.*, **54**, 161.

YOUNG, R.K., CAILLEAU, R.M., MACKAY, B & REEVES, W.J. (1974). Establishment of epithelial cell line MDA-MB-157 from metastatic pleural effusion of human breast carcinoma. *In Vitro*, **9**, 239.